# IL-17 Mediated Inflammation Promotes Tumor Growth and Progression in the Skin

**DOI:** 10.1371/journal.pone.0032126

**Published:** 2012-02-16

**Authors:** Donggou He, Hui Li, Nabiha Yusuf, Craig A. Elmets, Mohammad Athar, Santosh K. Katiyar, Hui Xu

**Affiliations:** 1 Department of Dermatology, University of Alabama at Birmingham, Birmingham, Alabama, United States of America; 2 Skin Disease Research Center, University of Alabama at Birmingham, Birmingham, Alabama, United States of America; 3 Birmingham VA Medical Center, Birmingham, Alabama, United States of America; City of Hope National Medical Center and Beckman Research Institute, United States of America

## Abstract

The mechanism for inflammation associated tumor development is a central issue for tumor biology and immunology and remains to be fully elucidated. Although IL-17 is implicated in association with inflammation mediated carcinogenesis, mechanisms are largely elusive. In the current studies, we showed that IL-17 receptor-A gene deficient (IL-17R-/-) mice were resistant to chemical carcinogen-induced cutaneous carcinogenesis, a well-established inflammation associated tumor model in the skin. The deficiency in IL-17R increased the infiltration of CD8+ T cells whereas it inhibited the infiltration of CD11b+ myeloid cells and development of myeloid derived suppressor cells. Inflammation induced skin hyperplasia and production of pro-tumor inflammatory molecules were inhibited in IL-17R-/- mice. We found that pre-existing inflammation in the skin increased the susceptibility to tumor growth, which was associated with increased development of tumor specific IL-17 producing T cells. This inflammation induced susceptibility to tumor growth was abrogated in IL-17R-/- mice. Finally, neutralizing IL-17 in mice that had already developed chemical carcinogen induced skin tumors could inhibit inflammation mediated tumor progression at late stages. These results demonstrate that IL-17 mediated inflammation is an important mechanism for inflammation mediated promotion of tumor development. The study has major implications for targeting IL-17 in prevention and treatment of tumors.

## Introduction

Immune surveillance mechanisms exist to recognize and eliminate tumor cells. Defects in immune surveillance are associated with tumor progression [1], [2], [3]. In anti-tumor immune responses, activated T cells infiltrate into tumors and destroy tumor cells either by cytotoxic effects or elicitation of inflammatory reactions that will involve other leukocytes in the eradication of tumors [4], [5]. However, chronic inflammation, an unsolved immune response, promotes tumor development [6], [7]. The infiltration of immune T cells within tumors, no matter at what stages of tumor development, is associated with beneficial prognosis [8]. In contrast, infiltration of granulocytes and macrophages has been considered as a promotion factor in tumor development [9], [10]. Intense inflammatory infiltrates comprised of large numbers of macrophages and granulocytes and high concentrations of inflammatory cytokines are characteristics of tumor promoting inflammation. They are considered to be the primary tumor promoting factors responsible for enhanced angiogenesis and cell growth [11].

IL-17 is an important cytokine responsible for inflammatory and autoimmune diseases [12], [13]. Although IL-17 producing cells are detected in cancer patients and tumor bearing animals [14], [15], studies which mostly use implanted tumor models show a controversial role of IL-17 in tumor development [14], [16]. Accumulating evidence indicates that IL-17 has tumor promoting effects, especially in the context of inflammation [17], [18], [19]. However, mechanisms for IL-17 mediated tumor promoting inflammation remains to be fully elucidated.

Dimethylbenz[*a*]anthracene (DMBA)-initiated and 12-O-tetradecanoylphorbol-13-acetate (TPA)-promoted cutaneous carcinogenesis is a well-established animal model for inflammation associated tumor development. The two-stage chemical carcinogenesis protocol includes single exposure to the mutagen DMBA followed by repeated application of the inflammation-inducing TPA. Skin lesions can be followed from premalignant papilloma formation to progression to carcinoma [20], [21], [22]. Although the effects of TPA are pleiotropic, its pro-inflammatory effects are crucial to tumor promotion. In mice that are deficient either in inflammatory cytokines IL-1β or TNF-α, DMBA/TPA induced carcinogenesis is inhibited [23], [24]. The arachidonic acid/cyclooxygenase (Cox) pathway of inflammation induction has a critical role in TPA induced tumor promotion [25], [26]. Additionally, S100A8/9, a group of calcium binding proteins, are critical for the development of chemical carcinogenesis [27], [28]. Mice deficient in the Receptor for Advanced Glycation End-products, a receptor for S100A8/A9, are resistant to DMBA/TPA induced carcinogenesis [27]. A defect in S100A9 results in increased infiltration of CD8+ T cells in tumors [29].

Our previous study showed that CD4+ T cells were primary IL-17 producing cells in DMBA/TPA induced cutaneous carcinogenesis [15]. Moreover, DMBA/TPA induced cutaneous carcinogenesis was enhanced in TLR4 deficient mice, which is associated with an increased level of IL-17 [30]. It has been reported that IL-23, a stimulatory cytokine for IL-17 production, promotes DMAB/TPA induced carcinogenesis [31] whereas it is inhibited in IL-17-/- mice [18]. However, mechanisms for IL-17 mediated effects on chemical carcinogenesis are largely unknown.

In the current studies, we examined DMBA/TPA induced cutaneous carcinogenesis in mice that were deficient in IL-17R, IL-12p35, or both IL-17R and IL-12p35. The effect of inflammation on tumor specific T cells and tumor growth and the role of IL-17 in tumor progression at the late stage were determined. Our findings indicate that IL-17 is required for DMBA/TPA induced carcinogenesis in the skin and that blockade of IL-17 suppresses inflammation mediated tumor development and progression.

## Results

### IL-17R deficient mice are resistant to chemical induced cutaneous carcinogenesis

**Figure 1:**
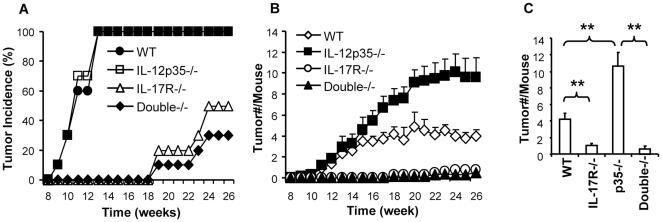
IL-17 receptor deficient mice are resistant to DMBA/TPA induced carcinogenesis. Mice were treated once with DMBA and then with TPA twice a week, as described in the Materials and Methods. Tumor growth was observed every week. A). Tumor incidence. B). Tumor multiplicity. C). The number of tumors was analyzed statistically at the end of experiments (26 weeks). The bar graph shows the mean number of tumors per mouse +/−SEM, *n* = 10, ** p<0.01. Data are representative of two independent experiments.

DMBA/TPA induced skin carcinogenesis was delayed and the incidence of tumors was greatly decreased in IL-17R-/- mice compared to wild type controls (Fig. 1a). Moreover, the tumor multiplicity was significantly reduced in IL-17R-/- mice (Fig. 1b and 1c). In p35-/- mice, which had a deficiency in IL-12 but not in IL-23, tumor multiplicity was significantly increased compared to wild type controls, although the incidence and kinetic of tumor development was not affected. However, mice that were deficient in both IL-17R and p35 genes (IL-17R/p35-/-), were resistant to tumor development in a similar manner to IL-17R-/- mice. These results indicate that IL-17 responses are required for DMBA/TPA induced carcinogenesis in the skin and that the effect of IL-17 on the chemical induced carcinogenesis is independent of IL-12 signals.

**Figure 2:**
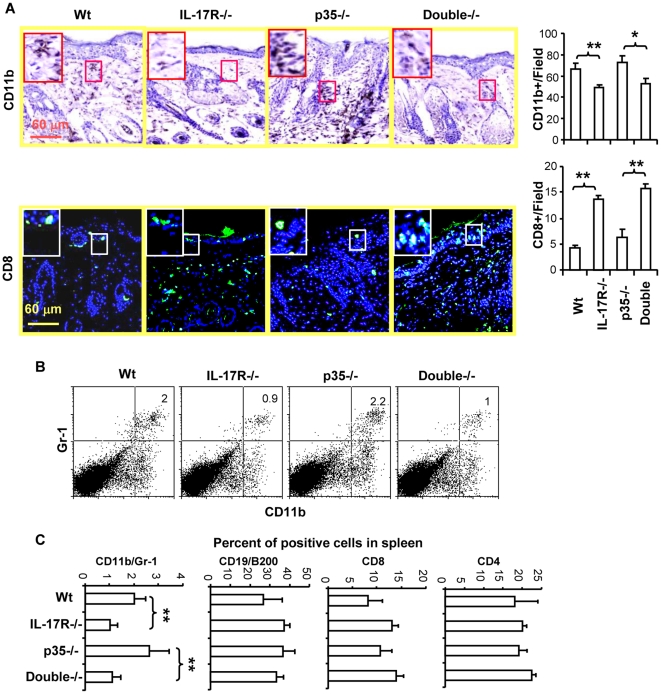
IL-17 has opposing effects on the infiltration of CD11b+ macrophages and CD8+ T cells in DMBA/TPA treated skin. Mice were treated with DMBA and TPA as described in figure 1 and skin tissues were harvested at the end of the experiment (26 weeks). A). Frozen tissue sections were stained with CD11b antibodies (brown) and counterstained with hematoxylin (blue, upper panels) or with anti-CD8 antibodies (green) and counterstained with a fluorescence dye DAPI (blue, lower panel). Photos were taken microscopically (20× objective). Numbers of positive cells were counted microscopically. Islets show the enlarged areas of circled squares with positive cells. Graphs show the mean number of positive cells per field +/− SEM, *n* = 10, * p<0.05, ** P<0.01. B). Gr-1 and CD11b double positive cells in spleens were stained with fluorescence labeled antibodies and positive cells were analyzed in a flow cytometer. The numbers in the right upper quadrant indicate the percent of positive cells in whole spleen cells. C). Statistical analysis of GR-1/CD11b+ (myeloid derived suppressor cells), CD19/B220+ (B cells), CD8+ and CD4+ T cells in spleens (mean +/− SEM, n = 8, ** P<0.01). Data are representative of two independent experiments.

The development of skin tumors in DMBA/TPA treated mice is dependent on TAP induced tumor promoting inflammation [15], [23]. To examine the effect of IL-17R deficiency on DMBA/TPA treated skin, mouse skin samples were harvested at the end of experiments (around 26 weeks) and subjected to immunohistochemical analysis. We found that the immigration of CD11b+ cells into the DMBA/TPA treated skin tissues was decreased (Fig. 2a). In contrast, the infiltration of CD8+ T cells was significantly increased in IL-17R-/- compared to wild type mice. The deficiency in p35 had little effect on the infiltration of CD11b+ cells and CD8+ T cells. However, similar to IL-17R-/- mice, IL-17R/p35-/- mice had an increased infiltration of CD8+ T cells and decreased infiltration of macrophages in the skin compared to p35-/- mice. The effect appeared to be selective on CD8+ T cells since the infiltration of CD4+ T cells was not significantly affected in IL-17R-/- and wild type mice (data not shown). The results implicate important effects of IL-17 on the inflammatory environment and CD8+ T cell responses in the skin during chemical induced carcinogenesis.

To determine whether the deficiency in IL-17 receptor has an effect on leukocytes in peripheral lymphoid organ, spleen cells from DMBA/TAP treated mice (26 weeks) were analyzed. Results showed that the percent and total number of CD4+ and CD8+ T cells and CD19/B200 double positive B cells were not significantly affected. However, CD1b/Gr-1 double positive myeloid cells are significantly decreased in the spleen of IL-17R-/- or p35/IL-17R-/- mice compared to p35-/- and wild type control animals (Fig. 2b and 2c).

### IL-17R deficiency inhibits TPA induced tumor promoting inflammation in the skin

**Figure 3:**
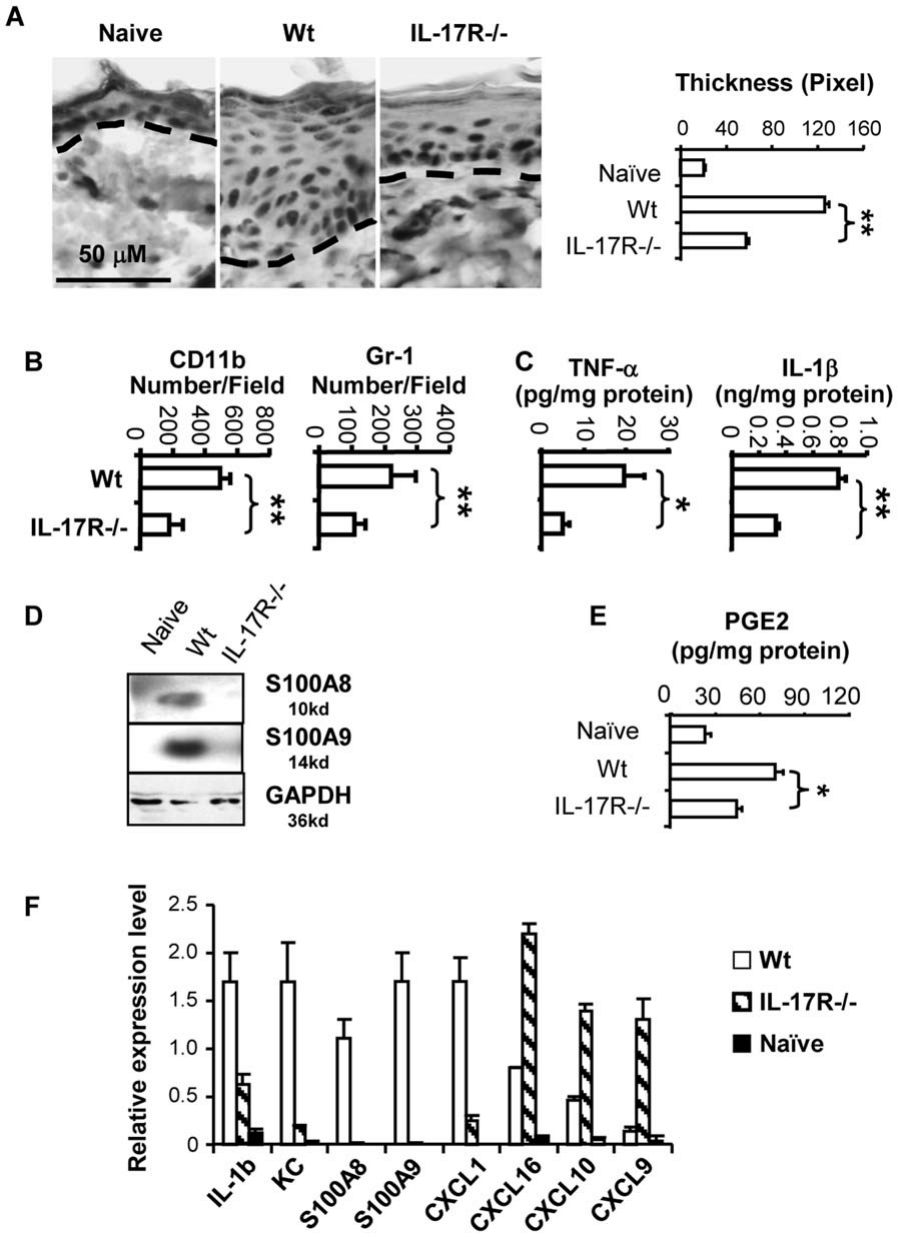
The deficiency in IL-17 receptor inhibits TPA induced tumor promoting inflammation in the skin. Mice were treated topically with TPA every other day for a total of three applications. Skin tissues were harvested 24 hours after the last treatment. A). Paraffin embedded tissue sections were stained with hematoxylin. Skin samples from naïve untreated mice served as controls. Dashed lines indicate the border between the epidermis and dermis. The thickness of epidermis was measured microscopically (n = 10). B). Frozen tissue sections were stained immunohistochemcially with antibodies. CD11b+ (monocytes/macrophages) and Gr-1+ (granulocytes) cells were counted microscopically (n = 6). C) Concentrations of cytokines in the skin lysates were measured by ELISA (n = 4). D). S100A8 and S100A9 proteins in the skin lysates were detected by Western blots using specific antibodies. E). Concentrations of PGE2 in the skin lysates were measured by a PGE2 EIA kit (n = 3). F). Expression levels of mRNA for chemokines and cytokines in the skin lysates were quantified by real time RT-PCR (n = 3). Data are representative of 2–3 independent experiments (mean +/− SEM, * P<0.05: ** P<0.01).

In the chemical carcinogenesis model, repeated treatments with TPA cause chronic inflammation which promotes tumor growth although no evidence shows that the treatment with TPA alone will induce carcinogenesis [15], [23]. Inflammation induced hyperplasia is an important mechanism for the development and progression of tumors [32], [33]. To test whether IL-17 regulates the development of TPA induced tumor promoting inflammation, mice were treated with TPA every other day for a total of three applications and skin samples were harvested 24 hours after the last treatment. We found that TPA induced epidermal hyperplasia was inhibited in IL-17R-/- mice compared to wild type controls (Fig. 3a). Additionally, we found that there were fewer CD11b+ macrophages and Gr-1+ granulocytes in the TPA treated skin of IL-17R-/- mice (Fig. 3b). Moreover, the levels of pro-tumor inflammatory cytokines IL-1β and TNF-α were reduced in the TPA treated skin of IL-17R-/- mice compared to wild type mice (Fig. 3c).

Western blot analysis showed that the TPA treatment induced the expression of S100A8/A9 in the skin of wild type mice whereas it was undetectable in IL-17R-/- mice (Fig. 3d). S100A8 and S100A9, two members of the S100 family of calcium binding proteins, are abundantly produced during acute and chronic inflammation and play an important role in inflammation associated tumor development [27], [28]. Similar findings were observed with Cox-2 and prostaglandin E_2_ (PGE2), a main substance converted by Cox-2, which plays important roles in TPA induced inflammation and tumor promotion in chemical induced carcinogenesis [25], [26]. The concentration of PGE2 was significantly higher in the TPA treated skin of wild type mice than in that of IL-17R-/- mice (Fig. 3e). Interestingly, the deficiency in IL-17R had different effects on chemokine expression in TPA treated skin tissues. Chemokines KC, CXCL1, and S100A8/A9, which regulate myeloid cell migration was reduced in IL-17R-/- mice. In contrast, chemokines CXCL16, CXCL10 and CXCL9, which are known for regulation of lymphocytes, were increased (Fig. 3f). Collectively, tumor promoting inflammation which is induced by the repeated treatment with TPA is suppressed in IL-17R-/- mice.

### Pre-treatment of skin with TPA enhances the development of tumor specific IL-17 producing T cells and increases the susceptibility of mice to tumor growth in an IL-17 dependent way

**Figure 4:**
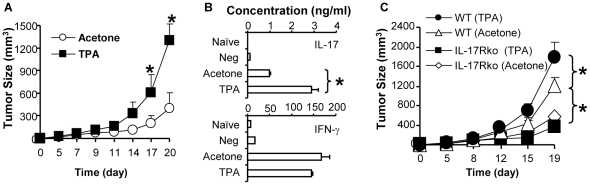
TPA induced promotion of tumor growth is abrogated in IL-17R-/- mice. A). TPA induced inflammation enhances tumor growth. Wild type C57Bl/6 mice were treated epicutaneously with TPA or acetone (vehicle controls) every other day for a total of 5 applications. The mice were then inoculated subcutaneously with EG7 tumor cells. Tumor growth was monitored (n = 5). B). TPA induced inflammation increases tumor specific IL-17 producing cells. At the end of experiments, the draining lymph node lymphocytes were collected from EG7 tumor bearing mice that were treated with TPA or acetone. The cells stimulated with OVA pulsed BM-DC for 4 days. Concentrations of cytokines in supernatants were measured by ELISA (n = 4). Naïve T cells with OVA pulsed BM-DC (Naïve) and immune T cells with BM-DC without OVA (Neg) served as controls in the cultures. C). TPA induced tumor promotion is abrogated in IL-17R-/- mice. Wild type or IL-17R-/- were epicutaneously treated with TPA or acetone every other day for a total of 5 applications. The mice were then inoculated with EG7 tumor cells and tumor growth was monitored (n = 5). Data are representative of 2–3 independent experiments (mean +/− SEM, * P<0.05).

Many cancers arise from the site of inflammation, which forms a microenvironment for tumor growth and progression [7]. In order to further examine roles of IL-17 in inflammation mediated tumor promoting microenvironment, mice were treated epicutaneously with TPA every other day for a total of 5 applications and then inoculated subcutaneously in the TPA treated skin area with an immunogenic mouse tumor cell line EG7 which is a mouse lymphoma cell line (EL4) transfected with ovalbumin (OVA). Results showed that the pre-treatment with TPA enhanced the growth of implanted EG7 tumors in wild type mice (Fig. 4a). It implicates that the pre-existing inflammation makes mice susceptible to tumor development. To examine whether inflammation regulated the development of tumor specific T cells, the draining lymph node lymphocytes were harvested from the mice which were pre-treated with TPA and then inoculated with EG7 tumors. The cells were then stimulated with OVA pulsed bone marrow derived dendritic cells (BM-DC) in cultures. Results showed that the pre-treatment with TPA induced a significantly higher level of tumor specific IL-17 producing T cells than those from control mice that were pre-treated with vehicle acetone (Fig. 4b). The production of IFN-γ was slightly but not significantly affected. To determine whether the increase in IL-17 producing lymphocytes was associated with the increased tumor growth in TPA treated mice, wild type and IL-17R-/- mice were treated with TPA or control acetone and then inoculated with EG7 tumor cells. We found that the treatment with TPA significantly enhanced tumor growth in wild type mice whereas it did not have a significant effect on tumor growth in IL-17R-/- mice (Fig. 4c). It is to note that the tumor growth in control IL-17R-/- mice was inhibited compared to that in control wild type mice as previously reported [34].

### Neutralization of IL-17 inhibits the progression of DMBA/TPA induced cutaneous carcinogenesis in mice that have already developed tumors

**Figure 5:**
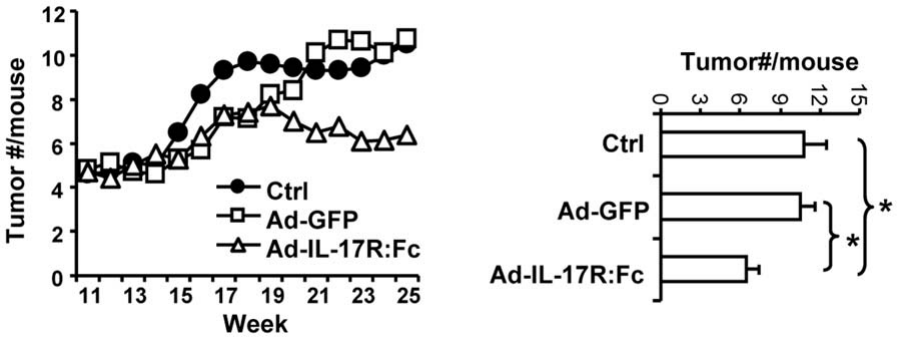
Neutralization of IL-17 inhibits the progression of tumors in DMBA/TPA induced cutaneous carcinogenesis. Wild type C57Bl/6 mice were topically treated once with DMBA and then twice weekly with TPA. All mice developed cutaneous tumors at 12 weeks. The mice were divided (10 mice/group) and treated intravenously with adenovirus encoding GFP (Ad-GFP), IL-17R:Fc (Ad-IL-17R:Fc) or left untreated (Ctrl) at week 12 and 16. All mice were continuously treated with TPA twice a week throughout the experiments. The tumor growth was monitored weekly. Bar graph shows the mean tumor number per mouse +/− SEM at the end of experiments (* P<0.05). Data are representative of 2 independent experiments.

Based on our results described above, which showed the suppression of TAP induced tumor promoting inflammation in IL-17R-/- mice, further experiments were to examine whether neutralization of IL-17 could inhibit inflammation associated tumor progression in mice which had already developed DMBA/TPA induced skin tumors. Mice with skin tumors (12 weeks after the DMBA/TPA treatment) were treated intravenously twice (week 12 and 16) with non-replicating adenovirus encoding neutralizing soluble IL-17R (Ad-IL-17R:Fc) or GFP as a control (Ad-GFP). The mice were kept with TPA treatment twice a week as described. Results showed that in the control mice which were left untreated, the number of skin tumors increased (Fig. 5). Neutralization of IL-17 with the administration of Ad-IL-17R:Fc significantly inhibited the progression of tumors compared to controls mice that were treated with Ad-GFP. The treatment with control Ad-GFP did not have a significant effect on tumor progression compared to mice that were left untreated (Fig. 5). The result implicates that neutralizing IL-17 can inhibit the progression of DMBA/TPA induced skin tumors.

## Discussion

The role of IL-17 in tumor development is controversial in implanted tumor models [14], [16], [35]. It appears that in induced tumor models which are associated with inflammation, IL-17 mediated inflammation has tumor promoting effects [18], [36]. However, mechanisms remain to be fully elucidated. We have showed here that mice deficient in IL-17R are resistant to DMBA/TPA induced carcinogenesis in the skin. The deficiency in IL-17R enhances CD8+ T cell mediated immune surveillance and inhibits tumor promoting inflammation in the skin. Furthermore, TPA induced inflammation promotes the susceptibility to the growth of implanted tumors in the skin and increases the development of tumor specific IL-17 producing T cells. However, this TPA induced tumor promoting effect is abrogated in IL-17R deficient mice. Finally, we show that neutralization of IL-17 inhibits the progression of DMBA/TPA induced skin carcinogenesis in mice which have already developed tumors. Our data demonstrates that IL-17 mediated inflammation promotes carcinogenesis in the skin and targeting IL-17 may be exploited to a new therapeutic strategy for tumors.

In DMBA/TPA induced skin carcinogenesis, IL-23 has been shown to enhance carcinogenesis. It is postulated that IL-23 mediated up-regulation of IL-17 production is a mechanism [31]. Similar to studies in IL-17-/- mice [18], we show that the deficiency in IL-17R render mice resistant to chemical induced skin carcinogenesis. Moreover, the IL-17R deficiency reverses the susceptibility of IL-12p35-/- mice to chemical carcinogenesis. It implicates that IL-17R mediated responses at least in part are attribute to increased tumor growth which is caused by IL-12 deficiency. However, mechanisms for the effect require further investigations. It is to note that in different tumor models, especially in implanted tumor models, IL-17 can have a protective effect against tumor development [37], [38], [39]. It suggests context dependent effects of IL-17 on tumors. Our data indicate that IL-17 has tumor promoting effects in inflammation associated tumor development in the DMBA/TPA model, which is inconsistent with reports showing that IL-17 promotes inflammation associated colon tumorigenesis [17], [36].

In DMBA/TPA induced chemical carcinogenesis, CD8+ T cells are primary effector cells and required for prevention of tumor development [15]. The opposite effect of IL-17 on the infiltration of CD11b+ myeloid cells and CD8+ T cells in the inflamed skin implicates a novel role of IL-17 in the regulation of immune surveillance during inflammation associated carcinogenesis in the skin. Interestingly, similar findings have been reported in implanted tumor models by our laboratory and others [34], [40]. Numerous clinical and laboratory studies show that the presence of macrophages is a hallmark of tumor promoting inflammation and is associated with a poor prognosis [9], [32] whereas the presence of CD8+ T cells is associated with a good prognosis [8], [41]. The effect of IL-17 on the production of chemokines has been well studied, which is considered to be responsible for its effects on the regulation of myeloid cell migration [42]. We have found that the deficiency in IL-17R reduces the expression of chemokines KC (human IL-8 analogue) and CXCL1 in TPA treated skin tissues, which are known to regulate the infiltration of myeloid cells in inflammation. In contrast, chemokines CXCL9, CXCL10 and CXCL16 which are known to regulate T cell migration are increased [43]. The differential effects of IL-17 on the expression of chemokines for myeloid cells and lymphocytes provide important clues for its effects on the regulation of myeloid cells and lymphocytes and may be important mechanisms for IL-17 mediated tumor promoting inflammation in DMBA/TPA induced skin tumors. Certainly, further studies are required to determine chemokine sources and to validate whether the effect of IL-17 on the chemokine production is associated with its effects on the regulation of leukocyte migration and development of skin tumors.

Depending on the functional status, myeloid cells including neutrophils and macrophages can have pro- or anti-tumor activities [4], [44], [45], [46]. However, it is a common feature that the number of myeloid derived suppressor cells (MDSC), which are defined as CD1b/Gr-1 double positive cells in mice, is increased in cancer patients and tumor bearing mice [47], [48], [49], [50]. MDSC are able to suppress anti-tumor immune responses and promote tumor growth [47], [50]. The decrease of MDSC provides a good explanation for the suppression of DMBA/TPA induced carcinogenesis in IL-17R-/- mice and may be an important mechanism for the role of IL-17 in the development of tumor promoting inflammation and immunosuppression. This is in accordance with our recent studies showing that IL-17 stimulates the function of MDSC in tumor bearing mice [34]. Additionally, MDSC are able to inhibit T cell infiltration of anti-tumor T cells in tumor sites [51]. The down regulation of MDSC in IL-17R-/- mice may contribute to the increase of CD8+ T cells in the inflamed skin.

The effect of inflammation on tumor promotion is mediated by complexes of mechanisms. We have found that the deficiency in IL-17R inhibits characteristics of tumor promoting inflammation which is mediated by the repeated treatment with TPA in the skin. Inflammation associated hyperplasia is a pro-tumor change which is observed in many inflammation associated tumorigenesis [32], [33]. Inflammatory infiltrates comprised of large numbers of macrophages and granulocytes and high concentrations of inflammatory cytokines are considered to be the primary factors responsible for enhanced angiogenesis and cell growth [9], [32]. TPA induced epidermal hyperplasia is inhibited in IL-17R-/- mice, which is associated with significantly reduced leukocyte infiltrations and levels of IL-1β and TNF-α. Literature shows that a deficiency in either IL-1β or TNF-α suppresses the development of chemical induced skin carcinogenesis [23], [24]. The reduction of IL-1β and TNF-α in IL-17R-/- mice may be part of mechanisms for the suppression of DMBA/TPA induced skin carcinogenesis.

Cox-2/PGE2 activities are significantly reduced in TPA treated skin of IL-17R-/- mice. Cox-2/PGE2 pathways have multiple effects on tumor development [50], [52], [53], [54]. Importantly, PGE2 not only promotes cell proliferation and tumor associated angiogenesis but also induces the development of MDSC [55], [56], [57]. Over expression of Cox-2 enhances whereas a defect in Cox-2 suppresses TPA induced inflammation and DMBA/TPA induced chemical carcinogenesis [25], [26]. Additionally, our results show that IL-17 is required for the expression of S100/A8/A9 in the TPA induced inflammation in the skin. S100A8/A9 proteins can induce the recruitment and differentiation of MDSC [58], [59]. A defect in *S100a9* inhibits the infiltration of MDSC whereas it results in increased infiltration of CD8+ T cells in tumors [29]. Collectively, the inhibition of TPA induced Cox-2/PGE2 activity and S100A8/A9 expression may be a critical mechanism for the reduction of MDSC, increases of CD8+ T cells in the skin, and the suppression of DMBA/TPA induced carcinogenesis in IL-17R-/- mice.

Many cancers arise from the site of inflammation, which forms a microenvironment for tumor growth and progression [7]. Increased levels of IL-17 and IL-17 producing T cells have observed in human and animal tumors [14], [16], [36]. A direct proof for an association of inflammation with induction of tumor specific IL-17 producing T cells and tumor development is not known. Our data show that pre-existing inflammation in the skin, which is induced by repeated treatment with TPA, increases the susceptibility of wild type mice to implanted tumors (Fig. 4). This effect is associated with a significantly increased level of tumor specific IL-17 producing T cells in the draining lymph nodes. In contrast, a significant effect on IFN-γ producing cells is not observed. Importantly, the promotion of tumor growth in TPA treated mice is abrogated in IL-17R-/- mice. Our study provides a strong support that inflammation induced increase of IL-17 producing T cells is a mechanism for the increased tumor growth and that blockade of IL-17 can inhibit inflammation mediated tumor promoting effects.

Although tumor promoting effects of inflammation have been well documented, it remains to be explored whether targeting inflammation can have therapeutic effects on existing tumors at late stages. Our data show that neutralization of IL-17 in mice that have already developed skin carcinogenesis induced by DMBA/TPA can inhibit tumor progression (Fig. 5). It indicates a role of IL-17 in inflammation mediated tumor progression at late phases and suggests that blocking IL-17 could have therapeutic effects. However, it is to note that neutralization of IL-17 can not eliminate tumors. Mechanisms for tumor development and tumor promoting inflammation are complicated. It is known that the immune system in tumor bearing host is suppressed. It is possible that IL-17 is just part of tumor promoting inflammation factors or that blockade of IL-17 diminishes the promoting effects of inflammation but can not restore the activity of tumor specific T cells to eliminate tumors. Interestingly, a recent report shows that IL-17 producing γδT cells contribute to the efficacy of anticancer chemotherapy. A deficiency in IL-17 responses inhibits the infiltration of cytotoxic T cells in the implanted colon tumors [60]. Similarly, in implanted melanoma model, Th17 cells promote the activity of cytotoxic T cells in tumors [61]. DMBA/TPA induced skin tumors are associated with persistent inflammation which may constitute a different microenvironment from that in implant tumors. It implicates that tumor treatment requires multifaceted strategies which will be dependent on tumor type, stage and microenvironment.

In summary, our studies have demonstrated that IL-17 is required for inflammation associated tumor development in DMBA/TPA induced skin carcinogenesis and IL-17 induces tumor promoting inflammation through multiple interactive mechanisms. TPA induced inflammation enhances the susceptibility to tumor growth, which is associated with an increased level of tumor specific IL-17 producing T cells. Blockade of IL-17 can abrogate the inflammation induced susceptibility to tumor growth and inhibit the progression of existing tumors in DMBA/TPA induced skin carcinogenesis. Given the important role of inflammation in tumor development, our studies implicate that targeting IL-17 may provide novel strategies for prevention and treatment of inflammation associated tumors.

## Materials and Methods

### Mice

IL-12p35-/- (C57BL/6 background) and wild type C57BL/6 mice were purchased from The Jackson Laboratory (Bar Harbor, Maine). IL-17R-/- mice on C57BL/6 background were provided by Amgen. IL-17R-/- mice were cross-bred to p35-/- mice to generate double knockout mice in our laboratory. The gene phenotype was routinely confirmed. All animal procedures were performed according to NIH guidelines and the protocols were approved by the Institute Animal Care and Use Committee of the University of Alabama at Birmingham (APN: 081008302).

### Cutaneous chemical carcinogenesis

Detailed protocols for DMBA/TPA induced carcinogenesis were reported previously [15]. In the current studies, mice were treated once with 100 µg DMBA (Sigma, St Louis, MO) in 100 µl acetone on the shaved back skin. One week later, the DMBA treated skin site was treated topically with 10 µg TPA in 100 µl acetone twice weekly. The development of cutaneous tumors was monitored every week. Tumors which were larger than 2 mm in diameter and were present for two consecutive weeks were counted. In compliance with IACUC requests, which were based on tumor multiplicity and size, mice were euthanized around 26 weeks.

To examine whether neutralization of IL-17 affected the progression of tumors in DMBA/TPA treated mice which had already developed skin tumors, mice were treated once with DMBA and then treated repeatedly with TPA as described above. At 12-14 weeks after the treatment with DMBA/TPA, all mice developed skin tumors. The mice were then divided in three groups with similar average tumor numbers per group (10 mice/group) and treated intravenously with non-replicating adenovirus encoding neutralizing soluble IL-17R (Ad-IL-17R:Fc) or GFP as a control (Ad-GFP)(10^9^ pfu/mouse) or left untreated. The treatment was repeated once 4 weeks later. This protocol has been reported in our previous studies to neutralize IL-17 and prevent autoimmune diseases in mice [62]. The mice remained to be treated with TPA twice a week and tumor progression was monitored once a week as described above.

### Examination of TAP induced tumor promoting inflammation in the skin

To examine TPA induced inflammation, back skin of mice was shaved and treated with TPA (10 µg in 100 µl acetone) every other day for a total of three applications. Skin samples were harvested 24 hours after the last treatment and subjected to analysis for inflammation.

To examine whether pre-existing chronic inflammation increased the susceptibility of mice to tumor growth, mice were topically treated on the shaved back with TPA (10 µg in 100 µl acetone) or vehicle control (acetone) every other day for a total of five applications. The mice were then implanted subcutaneously in the TAP treated area with a mouse lymphoma cells line EG7 (EL4 transfected with OVA, E.G7-OVA, CRL-2113, ATCC) at 4×10^6^/mouse one day after the last treatment with TPA. The growth of tumors was measured every three days by using an engineer's micrometer [34].

### Analysis of T cell cytokine production

The draining lymph nodes of EG7 tumor bearing mice were collected at the end of experiments and cell suspensions were prepared as described [34]. Bone marrow derived dendritic cells (BM-DC) were generated and pulsed with OVA for 24 hours as described in our previous report [34]. The lymph node cells (2×10^6^/ml) were stimulated with OVA-pulsed BM-DC (2×10^5^/ml) for 4 days. Culture supernatants were harvested and measured for concentrations of cytokines by ELISA as described [34]. Naïve T cells with OVA pulsed BM-DC (Naïve) and immune T cells with BM-DC without OVA (Neg) served as controls in the cultures.

### Immunohistochemical staining of skin tissues

Immunohistochemical staining of tissue sections was described in our previous studies [63]. Frozen tissue sections (5 µm) were applied for staining of CD8 and CD11b positive cells. Paraffin embedded sections (6 µm) were applied for staining of PCNA and Cox-2. The antibodies were purchased from Santa Cruz Biotechnology (Santa Cruz, CA), Cayman Chemical (Ann Arbor, MI) and BD-Biosciences (San Diego, CA). Pictures were taken microscopically (20× objective) with a digital camera (Olympus). Positive cells were counted in 10 fields of each group. Average numbers of positive cells per field were calculated and the difference between groups was analyzed statistically. The thickness of epidermis of skin sections was measured with the ImagePro Plus software.

### Analysis of PGE_2_ by enzyme immunoassay

The concentration of PGE2 in the TPA treated skin tissues was measured by using a prostaglandin E_2_ EIA kit according to the manufacturer's instructions (Cayman Chemical, Ann Arbor, MI). Briefly, skin samples were homogenized in the buffer provided by the kit and centrifuged at 11,000 rpm for 15 min. The concentration of PGE_2_ in supernatants was determined and normalized to the protein concentration in supernatants.

### Western blot

Western blots were conducted as reported in our previous studies [64]. To detect S100A8 and A9 proteins, skin tissues were harvested and lysed in the RIPA buffer. Equal amounts of proteins samples were loaded onto 7.5% SDS-PAGE and then transferred to nitrocellulose membranes. The membranes were probed with anti-S100A8, A9 or GAPDH antibodies. After wash, the blots were incubated with appropriate horseradish peroxidase-linked secondary antibodies. Protein bands were revealed by enhanced chemiluminescence (ECL) system (Pierce biotechnology, Rockford, IL). Blots were photographed with Bio-Rad gel documentation system and analyzed using Quantity one 1-D Analysis Software (Bio-Rad Laboratories, Hercules, CA).

### Detection of cytokines in skin tissues

Skin tissues were homogenized in the RIPA buffer. Concentrations of IL-1β and TNF-α in the lysates were measured by cytokine specific ELISA using commercial assay kits according to the manufacturer's instructions (Invitrogen). Concentrations of cytokines were normalized to protein concentrations in each sample.

### Real Time RT-PCR

The expression of mRNA for chemokines and inflammatory mediators was quantified by real time RT-PCR as described in our previous report [23]. Briefly, Skin tissues were homogenized in TRIzol and total RNA was isolated according to the manufacture's instructions (GibcoBRL). Real time TR-PCR was performed with iQ SYBRO Green Supermix Kit in a MyiQ real time qPCR system according to the manufacture's instructions (Bio-Rad). The expression level of cytokines was normalized to the house-keeping gene GAPDH in each sample. The sequences for primers were The sequences for primers were The sequences for primers were : CXCL9: forward, 5′-CCGCTGTTCTTTTCCTTTTG-3′, reverse, 5′-TCCCCCTCTTTTGCTTTTTC-3; S100A9: forward, 5′-TCATCGACACCTTCCATCAA-3′, reverse, 5′-GATCAACTTTGCCATCAGCA-3′; S100A8: forward, 5′-ACAATGCCGTCTGAACTGG-3′, reverse, 5′-CTCTGCTACTCCTTGTGGCTGTCT-3′; CXCL1: forward, 5′-GCTGGGATTCACCTCAAGAAC-3′, reverse, 5′-TGGGGACACCTTTTAGCATC-3′; CXC16: forward, 5′-ATGAGAAACAGCAAGATGACAG-3′, reverse, 5′-CAGTGAGGAAGAAGACAATGG -3′; CXCL10: forward, 5′-CGTCATTTTCTGCCTCATCC-3′, reverse, 5′-CAGACATCTCTGCTCATCATTC-3′; KC: forward, 5′-GATTCACCTCAAGAACATCCAG-3′, reverse, 5′-TGGGGACACCTTTTAGCATC-3′; IL-1β: forward, 5′-ACAGCAGCACATCAACAAGAG-3′, reverse, 5′-ATGGGAACGTCACACACCAG-3′; GApDH: forward, 5′-AATGGTGAAG GTCGGTGTGAAC-3′, reverse, 5′-GAAGATGGTGATGGGCTTCC-3′.

### Statistical analysis

All data are presented as means +/− SEM. Two-tailed Student's *t*-test was applied for statistical analysis with *P*<0.05 being considered statistically significant.
